# *Enterobacter* sp. AA26 gut symbiont as a protein source for Mediterranean fruit fly mass-rearing and sterile insect technique applications

**DOI:** 10.1186/s12866-019-1651-z

**Published:** 2019-12-24

**Authors:** Georgios A. Kyritsis, Antonios A. Augustinos, Spyridon Ntougias, Nikos T. Papadopoulos, Kostas Bourtzis, Carlos Cáceres

**Affiliations:** 1 Insect Pest Control Laboratory, Joint FAO/IAEA Programme of Nuclear Techniques in Food and Agriculture, A-1400 Vienna, Austria; 2 Laboratory of Entomology and Agricultural Zoology, Department of Agriculture Crop Production and Rural Environment, University of Thessaly, Phytokou St., 38446 N. Ionia, Magnisia Greece; 3 Laboratory of Wastewater Management and Treatment Technologies, Department of Environmental Engineering, Democritus University of Thrace, Vas Sofias 12, 67100 Xanthi, Greece

**Keywords:** Probiotics, *Ceratitis capitata*, Symbiotic bacteria, Gut microbiota, Nutrients, Biomass, Probiotic fermenter

## Abstract

**Background:**

Insect species have established sophisticated symbiotic associations with diverse groups of microorganisms including bacteria which have been shown to affect several aspects of their biology, physiology, ecology and evolution. In addition, recent studies have shown that insect symbionts, including those localized in the gastrointestinal tract, can be exploited for the enhancement of sterile insect technique (SIT) applications against major insect pests such as the Mediterranean fruit fly (medfly) *Ceratitis capitata*. We previously showed that *Enterobacter sp.* AA26 can be used as probiotic supplement in medfly larval diet improving the productivity and accelerating the development of the VIENNA 8 genetic sexing strain (GSS), which is currently used in large scale operational SIT programs worldwide.

**Results:**

*Enterobacter* sp. AA26 was an adequate nutritional source for *C. capitata* larvae, comprising an effective substitute for brewer’s yeast. Incorporating inactive bacterial cells in the larval diet conferred a number of substantial beneficial effects on medfly biology. The consumption of bacteria-based diet (either as full or partial yeast replacement) resulted in decreased immature stages mortality, accelerated immature development, increased pupal weight, and elongated the survival under stress conditions. Moreover, neither the partial nor the complete replacement of yeast with *Enterobacter* sp. AA26 had significant impact on adult sex ratio, females’ fecundity, adults’ flight ability and males’ mating competitiveness. The absence of both yeast and *Enterobacter* sp. AA26 (deprivation of protein source and possible other important nutrients) from the larval diet detrimentally affected the larval development, survival and elongated the immature developmental duration.

**Conclusions:**

*Enterobacter* sp. AA26 dry biomass can fully replace the brewer’s yeast as a protein source in medfly larval diet without any effect on the productivity and the biological quality of reared medfly of VIENNA 8 GSS as assessed by the FAO/IAEA/USDA standard quality control tests. We discuss this finding in the context of mass-rearing and SIT applications.

## Background

Gut-associated bacterial species are known to contribute to a wide range of services to their insect hosts including resistance to pathogenic microorganisms, protection against parasites and natural enemies, assistance in semiochemical communication, and most commonly the provision of nutrients [1]. The bacterial contribution to insects’ nutrition could be facilitated through i) extending the host digestive abilities (e.g. acquisition of digestive enzymes), ii) provisioning vitamins or other by-products of the bacterial metabolism, and in a more straight way by iii) digesting bacteria cells as a nutrient source [2, 3]. Hence, irrespective of their specific role, insects’ intestinal bacteria could be important elements of their host fitness.

Studies during the past decade investigated the gut microbiota of a major agricultural pest, the Mediterranean fruit fly (medfly) *Ceratitis capitata*. The characterization of medfly gut bacteria revealed the almost universal presence of *Enterobacteriaceae*, which seems to be the most abundant bacterial family in *C. capitata* gut microbiome. Despite the variation among studies, stemmed mainly by the experimentation with different medfly populations, developmental stages or methods used, the *Enterobacterales* species *Klebsiella, Enterobacter, Providencia, Pectobacterium, Pantoea, Morgonella* and *Citrobacter* are commonly isolated from medfly gut [4–6].

Following the identification of the gut microbial community, several recent studies attempted to determine the functional role of the gut bacteria by measuring their effects on medfly fitness. The probiotic effects of *Enterobacteriaceae* species were tested by manipulating their microbiota either with antibiotic depletion [7–9], or by feeding medfly with the isolated bacteria [5, 10–18]. *Enterobacteriaceae* microbiota were found to affect several medfly biological traits (e.g. by shortening immature development stages [14], increasing fecundity [15, 19, 20], extending survival [8, 11, 12], and improving male mating competitiveness [5, 11, 13] and female mating receptivity [13]). Although studies on the use of inactive bacterial cells as insect feed are limited, bacterial biomass can be served as single-cell protein to replace commercially-available proteinaceous sources and to enhance insect growth features, like pupal weight, e.g. by facilitating the bioconversion of the consumed leaf protein [4, 21].

Medfly is considered a quarantine pest that strongly affects the agricultural production, causing billions of economic losses worldwide. The wide spectrum of host plants and their broad geographical distribution necessitate an area-wide approach as the most appropriate strategy to manage medfly populations [22, 23]. The sterile insect technique (SIT) [24], as an integral part of AW-IPM programs, is being implemented over the last 4 decades against medfly [25], showing remarkable effectiveness worldwide. The main SIT principles consist of: i) the mass-production, ii) sterilization, and iii) the release of the sterilized insects in overwhelming ratios in relation to the wild population. The SIT success is largely dependent on the existence of a rearing protocol that ensures affordable and consistent production and release of sterile males of high biological quality, so that they can compete sufficiently with wild males for matings with wild females [26].

Currently, medfly is considered among the pests for which the SIT is used the most advanced. The introduction of new developments, like the releases of male-only through the establishment of the genetic sexing strains (GSS), and utilization of semiochemicals and other post-factory treatments to increase male mating competitiveness, have enhanced SIT efficiency against medfly [27–29]. Even though SIT is increasingly a cost-effective method for the population control of medfly, there are still some aspects that can be improved further to extend SIT perspectives, such as the reduction of the mass-rearing cost, which still consists a significant part of the overall operational costs [30].

The production of high quality larva of *Ceratitis capitata* demands large amounts of high protein source and other nutrients in order to achieve a stable and sustainable industrial production process. Protein for the larval diet is delivered using brewer’s or torula yeast (*Saccharomyces cerevisiae* and *Candida utilis*, respectively), which provide the essential amino acids required for larval development. Approximately 12% of the mass-rearing budget is allocated to the procurement of brewer’s or torula yeast (E. Ramirez, personal communication). The variability in the quality among different lots of yeast (different yeast sources), the limited number of reliable yeast-supplier companies, and the notable increased price in the last decade are additional elements to consider.

Considering the recent advances in the isolation and cultivation of medfly gut microbiota, as well as the potential role of some bacterial species (e.g. *Enterobacter* sp. AA26) as potential larval diet probiotics [14], we initiated this study to investigate whether *Enterobacter* sp. AA26 could partially or fully replace brewer’s yeast as a source of protein, meeting the nutritional demands required for the medfly larval diet and maintaining or even enhancing important biological “quality” traits in sterile flies, thus furthering the effectiveness of SIT programs.

## Materials and methods

### Medfly strains and rearing conditions

The experiments were conducted at the Joint FAO/IAEA Insect Pest Control Laboratory (IPCL), Seibersdorf, Austria, using the medfly Vienna 8 D53^−^ GSS, which carries the selectable markers white pupae (wp) [31] and temperature sensitive lethal (tsl) [32]. The flies were obtained from the El-Pino Guatemala mass-rearing facility and were reared at the IPCL for ten generations prior to their use in any experiment. Rearing was accomplished by keeping adults in two-side fine mesh cages and providing ad libitum water and adult diet, consisting of sugar and yeast hydrolysate at a 3:1 ratio. Eggs were deposited through the mesh and were collected from a water container placed under the mesh cover. Wild flies derived from field infested figs that were collected from the area of Volos, central Greece. Pupae recovered from the natural infested fruits were delivered to the IPCL. The colony was reared for five generations, providing bananas for oviposition, and the sixth generation adults (referred as wildish from now on) were used for the males’ competitiveness experiment. Both medfly colonies, Vienna 8 D53^−^ and the field collected population were kept at 22 °C, 65 ± 2% RH and 14 h L: 10 h D.

### Inactive *Enterobacter* sp. AA26 biomass production

The medfly gut symbiont *Enterobacter* sp. AA26 was grown aseptically at 24 °C in 1 L laboratory-scale bioreactors of 0.6 L working volume each, which were fed with Luria-Bertani (LB) broth and operated under the fill and draw mode. An air pump was used for each bioreactor to achieve adequate aeration, i.e. dissolved oxygen values above 4 mg/L, whereas the bacterial culture was continuously agitated. Bacterial biomass collection was achieved by centrifugation at 4000 g for 10 min and storage of the obtained biomass at -80 °C until being delivered to the IPCL under iced conditions.

### Larval diet preparation

**Fig. 1:**
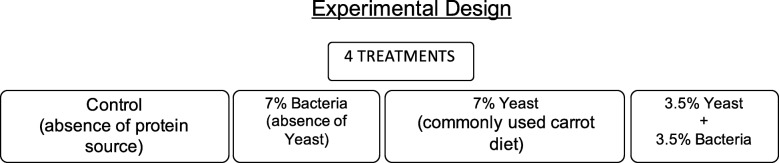
Experimental plan followed for the estimation of *Enterobacter* sp. AA26 potential as protein source substitute

Using a carrot larval diet containing 7% brewer’s yeast (supplier: Mraz Agro CZ Ltd) and medium scale rearing conditions, we tried to explore the effect of yeast replacement with bacteria on the development and the life history parameters of medfly. Specifically, we studied in the Vienna D53^−^ GSS, the effects of the: a) full yeast replacement with *Enterobacter* sp. AA26 biomass (7% bacterial biomass instead of 7% brewer’s yeast), b) partial yeast replacement (3.5% brewer’s yeast plus 3.5% *Enterobacter* sp. AA26 biomass), and c) absence of both brewer’s yeast and bacterial biomass from the larval diet as control (Fig. 1). In order to achieve a uniform texture among the treatments, a small quantity of corncob, a bulking agent with negligible nutritional profile [33], was added to all treatments, excluding the treatment that contained 7% brewer’s yeast in which the larval diet presents uniform texture and therefore a bulking agent was not required (Table 1). The bacterial biomass collected was placed at 60 °C until obtaining a completely dry material of biomass (approximately 48 h). The dry bacterial biomass was weighted and the respective amount was incorporated in each carrot diet treatment. Eggs collected during a 6 h interval were placed on moist filter paper before putting on the larval diet. The larval development of the flies that used to evaluate immature survival, immature development, pupal weight, adult demography, flight ability, longevity under stress and males mating competitiveness took place in round size, 70 × 15 mm, petri dishes (300 eggs were seeding in 150 g carrot diet in each petri dish). The number of individual replicates used in each experiment are given below in the respective M&M paragraph).

**Table 1:** Diet formulas used to evaluate the potential of brewer’s yeast replacement with *Enterobacter* sp. AA26 in medfly larval diet

Ingredients	Diet formulas			
	Control	7% Yeast	7% Bacteria	3.5% Yeast + 3.5% Bacteria
*Enterobacter* sp. AA26	–	–	70 g	35 g
Brewer’s Yeast	–	70 g	–	35 g
Corncob	2.0 g	–	2.0 g	1.0 g
Carrot powder	150 g	150 g	150 g	150 g
HCL	8.0 ml	8.0 ml	8.0 ml	8.0 ml
Na Benzoate	2.5 ml	2.5 ml	2.5 ml	2.5 ml
Nipagin	2.0 g	2.0 g	2.0 g	2.0 g
Water	770 ml	770 ml	770 ml	770 ml

### Pupal-adult recovery and developmental times

Pupae were collected daily (at 11:00) and transferred to a petri dish until emergence in order to record the survival and the developmental times during the immature stages. The adult emergence was recorded daily, at 11:00, as well. Three replicates (round size, 70 × 15 mm, petri dishes with 150 g carrot diet) per treatment were performed, with 300 eggs each. In view that the control treatment resulted in extremely low recovery rates, we chose to exclude the control treatment from the evaluation of the following quality parameters (pupal weight, fecundity, flight ability, longevity under water and food deprivation, males mating competitiveness).

### Pupal weight

Three petri dishes with 300 eggs each (as described above) were set up for each one of the different larval diets. All pupae that recovered the same day from the same larval treatment (3 petri dishes) were uniformly mixed. Pupal weight was determined by individually weighing 100 (50 males and 50 females) randomly selected pupae per treatment, (7% brewer’s yeast, 7% *Enterobacter* sp. AA26 biomass, 3.5% brewer’s yeast & 3.5% *Enterobacter* sp. AA26 biomass), 2–3 days before adult emergence.

### Fecundity

Three petri dishes with 300 eggs each (as described above) were set up for each one of the different larval diets. All pupae that recovered from the same larval treatment (3 petri dishes) were uniformly mixed. Within 6 h from adult emergence, one randomly selected female and two males, to ensure female insemination, were placed in 200 cm^3^ volume rectangular plexiglass cages. Water and standard adult diet were provided ad libitum. One side of the cages were covered with fine mesh that was used by females for egg laying. The eggs were collected from a black filter paper placed under that side of the cage. Female fecundity was recorded as daily egg count until the 16th day of adult age. We chose to evaluate this narrow period of the females’ life and not the whole lifespan considering that the period of egg collections in mass-rearing facilities is strictly up to 15 days of age. A total of 20 replicates were run for each of the three different larval treatments (7% brewer’s yeast, 7% *Enterobacter* sp. AA26 biomass, and 3.5% brewer’s yeast & 3.5% *Enterobacter* sp. AA26 biomass).

### Flight ability

Three petri dishes with 300 eggs each (as described above) were set up for each one of the different larval diets (7% brewer’s yeast, 7% *Enterobacter* sp. AA26 biomass, 3.5% brewer’s yeast and 3.5% *Enterobacter* sp. AA26 biomass). All pupae that recovered from the same larval treatment (3 petri dishes) were uniformly mixed. Fifty male and 50 female pupae, brown and white respectively in the Vienna D53^−^ GSS, were randomly selected and placed within a ring of paper centered in the bottom of a Petri dish. A black plexiglass tube was adjusted over the Petri dish. The inside of the tube was lightly coated with unscented talcum powder to prevent the flies from walking out. Flies were periodically removed from the vicinity of the tubes to minimize fly-back or fall-back into the tubes. The experiment was conducted at 26 °C, 65% RH, 14 h L: 10 h D and 1500 lx light intensity. Three replicates with 100 pupae each were set up per treatment (7% brewer’s yeast, 7% *Enterobacter* sp. AA26 biomass, 3.5% brewer’s yeast and 3.5% *Enterobacter* sp. AA26 biomass). The procedure is described in detail in [34].

### Longevity under food and water deprivation

Three petri dishes with 300 eggs each (as described above) were set up for each one of the different larval diets. All pupae that recovered the same day from the same larval treatment (3 petri dishes) were uniformly mixed. Two days before emergence, 100 white and 100 brown randomly selected pupae from each treatment (7% brewer’s yeast, 7% *Enterobacter* sp. AA26 biomass, 3.5% brewer’s yeast & 3.5% *Enterobacter* sp. AA26 biomass) were placed in wire-screen covered, well plates, to measure longevity under stress (food and water deprivation). Each pupa was hosted individually in a well, sized 1x1x1cm. Plates were kept in the dark at 22 ± 1 °C and 55 ± 5% RH, and were examined every 6 h in order to record the time of emergence and death for each insect.

### Male mating competitiveness

The mating competitiveness ability of the Vienna 8 D53^−^ males derived from three of the larval diet treatments (7% brewer’s yeast, 7% *Enterobacter* sp. AA26 biomass, 3.5% brewer’s yeast & 3.5% *Enterobacter* sp. AA26 biomass) was tested against wildish males when competing for wildish females. Three petri dishes with 300 eggs each (as described above) were set up for each one of the different larval diets. All pupae that recovered the same day from the same larval treatment (3 petri dishes) were uniformly mixed. Adult flies were sorted by sex within 24 h from emergence and were kept in round Plexiglass cages. Flies had ad libitum access to water and adult diet consisting of sugar and yeast hydrolysate at a 3:1 ratio. The wildish flies (males and females) were tested when 7–11 days old and the Vienna 8 D53^−^ males when 4–6 days old. One to two days before emergence the Vienna 8 D53^−^ males were irradiated applying 120 Gy in a Gammacell 220 irradiator. Mating tests were conducted in the IPCL greenhouse under controlled temperature and humidity conditions (26 ± 1 °C, 45–55% RH). One potted *Citrus* sp. tree was placed into each of the 2.0 × 1.6 × 1.9 cm sized field cages. The day before test, both wildish and Vienna 8 D53^−^males were marked on the thorax with a yellow or red dot of a non-toxic dye. The color and the field cages used for the experiment were rotated between Vienna 8 D53^−^ and wildish males to eliminate any bias. On the experimental days, 50 males (25 Vienna 8 D53^−^ and 25 wildish) and 25 females were released into each of the field cages, at 07:30 and 09:00 respectively. The field cages were inspected every 15 min until 15:00. Once a couple was detected, it was placed in a transparent vial where it was maintained until the end of the copulation. A total of 6–7 replicates were performed for each treatment (7% brewer’s yeast, 7% *Enterobacter* sp. AA26 biomass, 3.5% brewer’s yeast & 3.5% *Enterobacter* sp. AA26 biomass).

### Statistical analysis

Data analyses were performed using SPSS 23.0 (SPSS Inc., Chicago, IL, U.S.A.). The effect of yeast replacement with bacteria (partially or completely) on pupae and adult recovery rates were estimated using generalized linear modeling techniques. A hierarchical structure was used by nesting within replications. Power analysis was used to infer the effects of yeast replacement on flight ability. Kaplan-Meier estimators of immature developmental times (pupation day, pupal stage duration and total immature stages duration) were calculated to determine the effects of yeast replacement with bacteria on these parameters. Pairwise comparisons among the three treatments (7% brewer’s yeast, 7% *Enterobacter* sp. AA26 biomass, 3.5% brewer’s yeast & 3.5% *Enterobacter* sp. AA26 biomass) were conducted using the log-rank (Mantel-Cox) test. The effect of brewer’s yeast replacement by *Enterobacter* sp. AA26 biomass on pupal weight, adult sex ratio, fecundity and male mating competitiveness ability was assessed by ANOVA (Tukey’s HSD test for pairwise comparisons), whereas the effect on adult ability to survive under stress conditions was determined by Cox regression analysis.

## Results

### Effect of brewer’s yeast replacement by *Enterobacter* sp. AA26 biomass on pupa and adult recovery

**Fig. 2:**
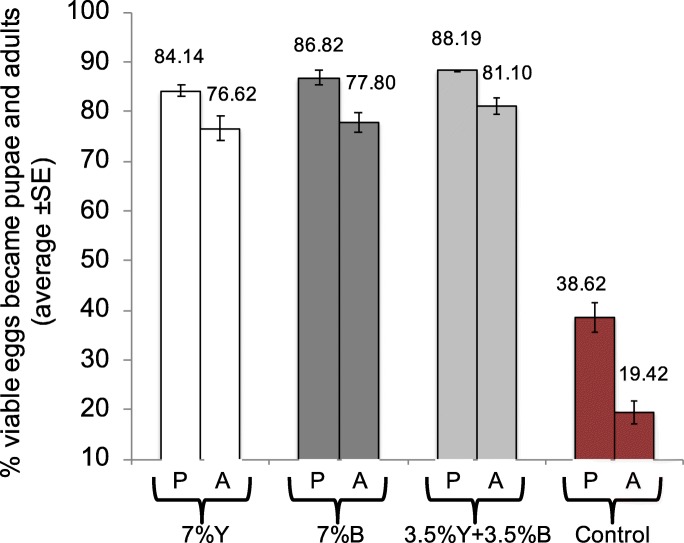
Immature stages survival (P: pupae recovery, A: adult recovery)

**Fig. 3:**
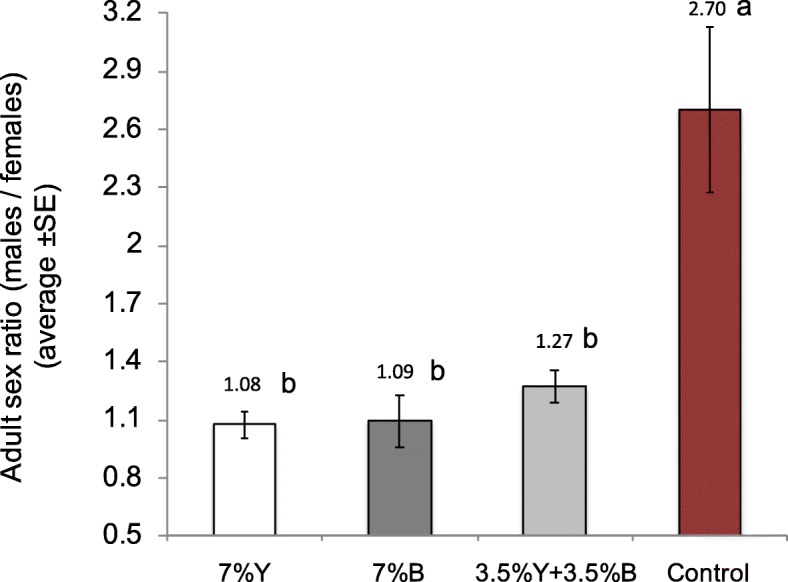
Adult sex ratio determination

The analysis on the proportion of the viable (hatched) eggs that developed into pupae and adults revealed that brewer’s yeast (Y) replacement with *Enterobacter* sp. AA26 biomass (B) indicated significant effects on both pupae and adult recovery rates (Fig. 2; Wald’s t-test t = 589.18, 685.38, df = 3, *P* < 0.0001, respectively). Partial yeast replacement with bacterial biomass (3.5% Y+ 3.5% B) increased the pupal and adult recovery rates over the only brewer’s yeast (7%Y) containing diet (Wald’s t-test t = 4.07, 6.01, df = 3, *P* = 0.044, 0.014, respectively). Full yeast replacement with bacterial biomass (7% B) led to increased pupae and adult recovery rates over the yeast treatment (7% Y), although this difference was not significant (Wald’s t-test t = 0.62, 0.33, df = 1, *P* = 0.43, 0.57). The higher recovery rates recorded for partially yeast replacement (3.5% Y + 3.5% B) were not significant compared to full yeast replacement treatment (7% B) (Wald’s t-test t = 1.59, 3.66, df = 1, *P* = 0.21, 0.06, for pupae and adults’ recovery, respectively). The absence of both yeast and bacterial biomass in the larval diet detrimentally reduced the recovered pupae and adults over all the other treatments (Wald’s t-test t = 109.66, 122.59, 132.97, df = 1, *P* < 0.001 for pupae and Wald’s t-test t = 142.07, 153.65, 181.21, df = 1, P < 0.001 for adults, over 7% Y, 7% B and 3.5% Y + 3.5% B, respectively). The absence of both yeast and bacterial biomass from the larval diet (control treatment) resulted in extremely high sex ratios in favor of the males compared to the other treatments (F = 11.57, df = 3, 11, *P* = 0.003). On the other hand, the provision of yeast, bacterial biomass or both (7% Y, 7% B, 3.5% Y + 3.5% B) had a similar effect on sex ratio (Fig. 3).

### Effect of brewer’s yeast replacement with *Enterobacter* sp. AA26 biomass on medfly immature development

#### Egg to pupa

**Fig. 4:**
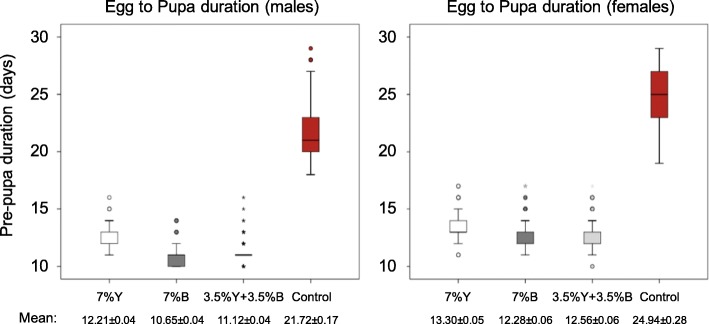
Egg to pupa developmental duration of the four protein source treatments

Figure 4 (and Additional file 1A) depicts the accumulated duration of egg and larva stages. The partial brewer’s yeast replacement with *Enterobacter* sp. AA26 biomass (3.5% Y + 3.5% B) resulted in significantly reduced pre-pupal duration for both males and females (Table 2) compared to the 7% Y treatment. In addition, the full brewer’s yeast replacement (7% B) led to significant earlier pupation compared not only to the 7% Y, but also to 3.5% Y + 3.5% B treatment, for both sexes. The egg to pupal developmental time was significantly longer in the control treatment (no yeast and no bacterial biomass provision) compared to all other treatments, irrespective of the sex (Table 2).

**Table 2:** Brewer’s yeast replacement with *Enterobacter* sp. AA26 biomass and egg to pupa developmental duration

Treatment	N	Mean (days) ± SE	Kaplan-Meier / log-rank (Mantel-Cox)
Males			
7% Y	331	12.21 ± 0.041	7% Y vs 3.5% Y + 3.5% B: x^2^ = 289.85, *P* < 0.001
7% B	358	10.65 ± 0.039	7% B vs 3.5% Y + 3.5% B: x^2^ = 81.48, *P* < 0.001
3.5% Y + 3.5% B	375	11.12 ± 0.036	7% Y vs 7% B: x^2^ = 422.73, P < 0.001
Control	208	21.72 ± 0.171	Control vs 3.5% Y + 3.5% B: x^2^ = 585.11, P < 0.001
			Control vs 7% Y: x^2^ = 545.19, P < 0.001
			Control vs 7% B: x^2^ = 546.65, P < 0.001
Females			
7% Y	311	13.30 ± 0.055	7% Y vs 3.5% Y + 3.5% B: x^2^ = 69.55, P < 0.001
7% B	327	12.28 ± 0.058	7% B vs 3.5% Y + 3.5% B: x^2^ = 11.12, *P* = 0.001
3.5% Y + 3.5% B	297	12.56 ± 0.061	7% Y vs 7% B: x^2^ = 131.65, P < 0.001
Control	94	24.94 ± 0.276	Control vs 3.5% Y + 3.5% B: x^2^ = 309.37, P < 0.001
			Control vs 7% Y: x^2^ = 321.70, P < 0.001
			Control vs 7% B: x^2^ = 325.32, P < 0.001

#### Pupal stage

**Fig. 5:**
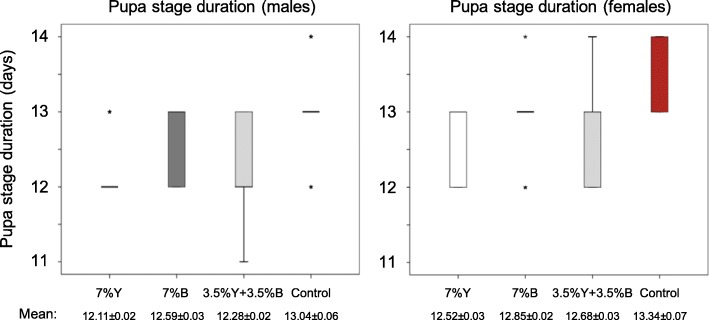
Pupa stage developmental duration of the four protein source treatments

Brewer’s yeast replacement seems to exert the opposite effect on pupal stage duration than in egg to pupa developmental duration. Specifically, partial replacement (3.5% Y + 3.5% B) with *Enterobacter* sp. AA26 significantly increased the pupa stage duration compared to the 7% Y treatment, for both sexes (Fig. 5, Additional file 1B, Table 3). Moreover, full replacement (7% B) significantly increased pupae developmental duration compared not only to the 7% Y, but also to the partial replacement (3.5% Y + 3.5% B), for both males and females. The control treatment (absence of both yeast and bacteria from the larval diet) significantly prolonged the pupal developmental duration compared to all other treatments, irrespective of the sex (Table 3).

**Table 3:** Brewer’s yeast replacement with *Enterobacter* sp. AA26 biomass and pupal stage duration

Treatment	N	Mean (days) ± SE	Kaplan-Meier / log-rank (Mantel-Cox)
Males			
7% Y	302	12.11 ± 0.018	7% Y vs 3.5% Y + 3.5% B: x^2^ = 27.27, P < 0.001
7% B	319	12.59 ± 0.027	7% B vs 3.5% Y + 3.5% B: x^2^ = 65.77, P < 0.001
3.5% Y + 3.5% B	345	12.28 ± 0.024	7% Y vs 7% B: x^2^ = 154.99, P < 0.001
Control	111	13.04 ± 0.056	Control vs 3.5% Y + 3.5% B: x^2^ = 134.06, P < 0.001
			Control vs 7% Y: x^2^ = 203.71, P < 0.001
			Control vs 7% B: x^2^ = 53.41, P < 0.001
Females			
7% Y	283	12.52 ± 0.029	7% Y vs 3.5% Y + 3.5% B: x^2^ = 14.98, P < 0.001
7% B	295	12.85 ± 0.023	7% B vs 3.5% Y + 3.5% B: x^2^ = 21.01, P < 0.001
3.5% Y + 3.5% B	273	12.68 ± 0.029	7% Y vs 7% B: x^2^ = 69.85, P < 0.001
Control	41	13.34 ± 0.075	Control vs 3.5% Y + 3.5% B: x^2^ = 55.49, P < 0.001
			Control vs 7% Y: x^2^ = 71.87, P < 0.001
			Control vs 7% B: x^2^ = 46.08, P < 0.001

#### Egg to adult

**Fig. 6:**
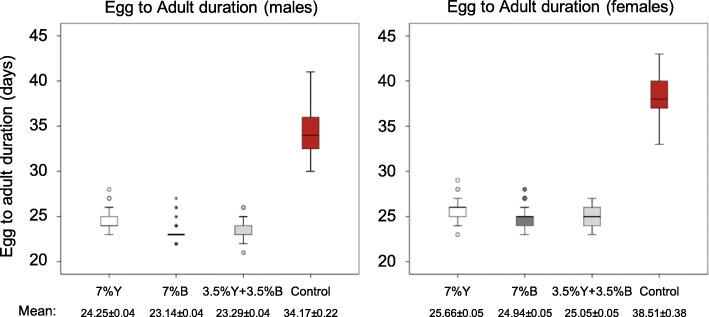
Total duration of immature stages (egg to adult) of the four protein source treatments

The overall (egg + larva + pupa) immature developmental time data are depicted in Fig. 6 (and Additional file 1C). In respect to males, full brewer’s yeast replacement with *Enterobacter* sp. AA26 (7% B) significantly accelerated adult emergence compared to 3.5% Y + 3.5% B. The effect was even more pronounced when 7% B fed males compared with 7% Y fed ones. Moreover, 3.5% Y + 3.5% B fed males also completed the immature development significantly faster than the males fed on 7% Y (Table 3). Regarding females, although the immature developmental duration of the 7% B fed larvae was shorter than 3.5% Y + 3.5% B fed, this difference was not statistically significant. On the other hand, 7% Y fed females showed significantly increased immature stages duration compared to both 3.5% Y + 3.5% B and 7% B treatments. The flies of both sexes that fed on the control treatment (absence of both yeast and bacteria from the larval diet) completed the immature development significantly later than all other treatments tested (7% Y, 7% B, 3.5% Y + 3.5% B) (Table 4).

**Table 4:** Brewer’s yeast replacement with *Enterobacter* sp. AA26 biomass and Immature stages developmental duration

Treatment	N	Mean (days) ± SE	Kaplan-Meier / log-rank (Mantel-Cox)
Males			
7% Y	302	24.25 ± 0.044	7% Y vs 3.5% Y + 3.5% B: x^2^ = 204.43, P < 0.001
7% B	319	23.14 ± 0.039	7% B vs 3.5% Y + 3.5% B: x^2^ = 7.28, *P* = 0.007
3.5% Y + 3.5% B	345	23.29 ± 0.037	7% Y vs 7% B: x^2^ = 252.11, P < 0.001
Control	111	34.17 ± 0.225	Control vs 3.5% Y + 3.5% B: x^2^ = 381.59, P < 0.001
			Control vs 7% Y: x^2^ = 356.90, P < 0.001
			Control vs 7% B: x^2^ = 349.70, P < 0.001
Females			
7% Y	283	25.66 ± 0.053	7% Y vs 3.5% Y + 3.5% B: x^2^ = 56.02, P < 0.001
7% B	295	24.94 ± 0.049	7% B vs 3.5% Y + 3.5% B: x^2^ = 3.15, *P* = 0.076
3.5% Y + 3.5% B	273	25.05 ± 0.054	7% Y vs 7% B: x^2^ = 88.03, P < 0.001
Control	41	38.51 ± 0.383	Control vs 3.5% Y + 3.5% B: x^2^ = 162.49, P < 0.001
			Control vs 7% Y: x^2^ = 165.96, P < 0.001
			Control vs 7% B: x^2^ = 176.98, P < 0.001

As the absence of both brewer’s yeast and *Enterobacter* sp. AA26 bacteria resulted in extremely low recovery rates and much longer overall immature developmental times, a range of parameters such as pupal weight, fecundity, flight ability, longevity under stress conditions and males’ mating ability, were evaluated only for the three treatments, i.e. 7% Y, 7% B and 3.5% Y + 3.5% B.

### Effect of brewer’s yeast replacement with *Enterobacter* sp. AA26 on pupal weight

**Fig. 7:**
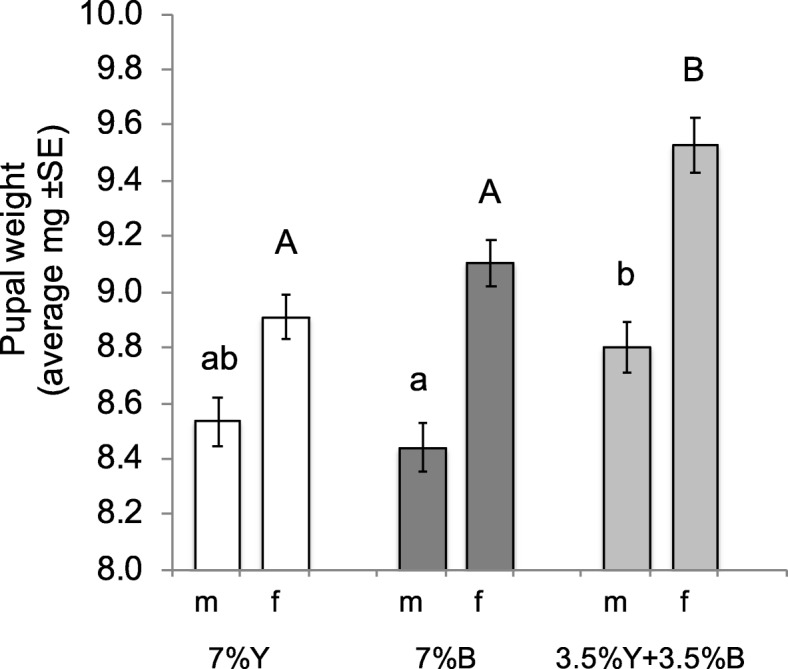
Males’ and females’ pupal weight of the flies fed on the three different protein source diets (m: males, f: females). Columns marked on the top with the same letter are not significantly different (*P* > 0.05)

Brewer’s yeast replacement with *Enterobacter* sp. AA26 biomass significantly affected the pupal weight of both males and females (F = 4.46, df = 2149, *P* = 0.01 and F = 13.11, df = 2149 *P* < 0.001, respectively), (Fig. 7). Tukey’s HSD test for pairwise comparisons among the tested diets (7% Y, 7% B, 3.5% Y + 3.5% B) revealed that the male pupae of the combined yeast and bacteria treatment (3.5% Y + 3.5% B) were heavier compared to those of the only bacteria (7% B) treatment, but did not differ from those of the standard diet (7% Y). Regarding females, the combined provision of yeast plus bacteria (3.5% Y + 3.5% B) resulted in significantly heavier pupae compared to both 7% B and 7% Y treatments. The only bacteria or only yeast provision (7% B or 7% Y, respectively) had the same effect on female pupal weight.

### Effect of brewer’s yeast replacement with *Enterobacter* sp. AA26 on fecundity

**Fig. 8:**
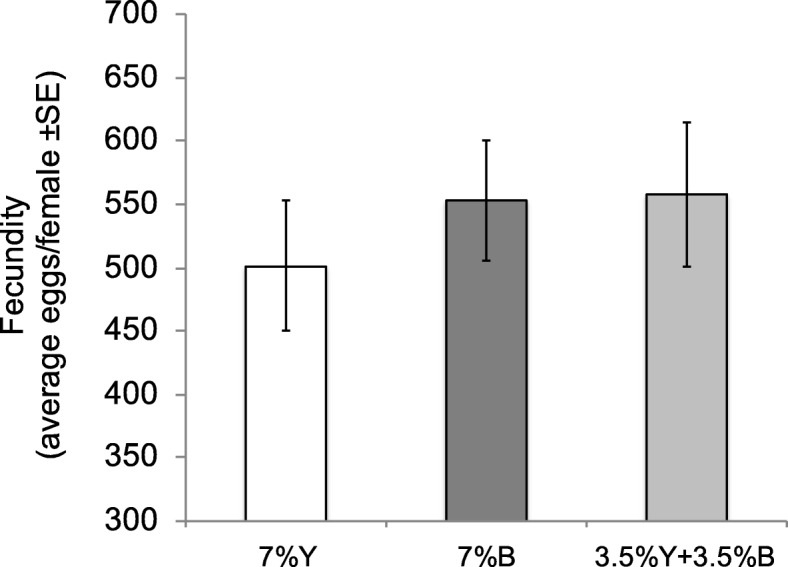
Females’ fecundity of the flies fed on the three protein source diets

Figure 8 shows the egg production recorded for the females of the three larval diet treatments (7% Y, 7% B, 3.5% Y + 3.5% B) until the age of 16 days. ANOVA analysis did not detect significant differences among 7% Y, 7% B and 3.5% Y + 3.5% B diets (F = 0.37, df = 2,59, *P* = 0.69). However, a trend for increased fecundity in bacterial diets, either 7% B or 3.5% Y + 3.5% B, compared to the only yeast treatment (7% Y), was recorded.

### Effect of brewer’s yeast replacement with *Enterobacter* sp. AA26 on flight ability

**Fig. 9:**
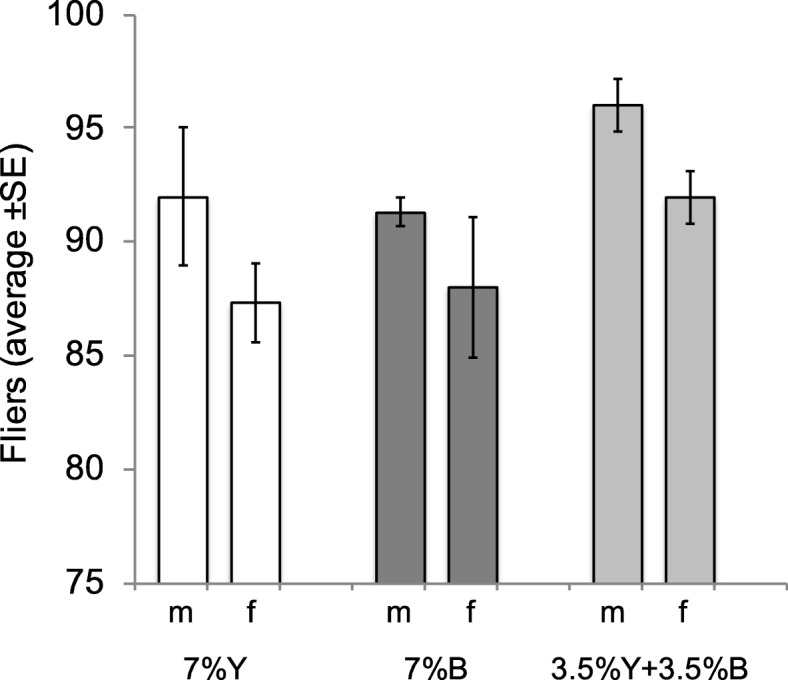
Flight ability of the flies fed on the three protein source diets (m: males, f: females)

Logistic regression analysis revealed that yeast replacement was not a significant predictor of flight ability (Wald’s t-test t = 4.53, df = 2, *P* = 0.104). However, our study was slightly underpowered in detecting significant differences within this setting as revealed by post-hoc power analysis. This fact can be considered as a limitation of our study. The flight ability of males was significantly higher than that of females in all three treatments (Wald’s t-test t = 4.37, df = 1, *P* = 0.036, Fig. 9).

### Effect of brewer’s yeast replacement with *Enterobacter* sp. AA26 on longevity under stress conditions

**Fig. 10:**
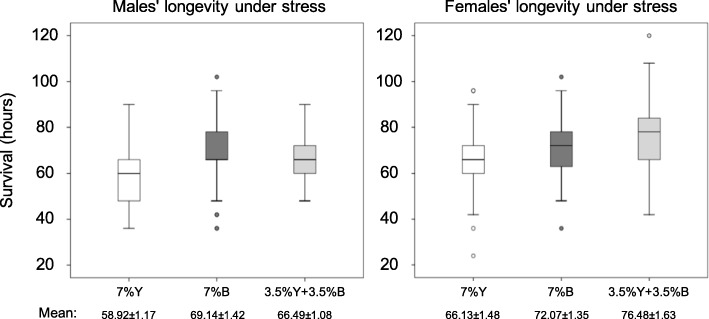
Survival under stress conditions of males and females on the three protein source diets (m: males, f: females)

Cox regression analysis, with larval diet treatments (7% Y, 7% B, 3.5% Y + 3.5% B) and sex as covariates, revealed that both diet (Wald’s t-test t = 14.87, df = 2, *P* = 0.001) and sex (Wald’s t-test t = 19.67, df = 1, *P* < 0.001) were significant predictors of adult survival under water and food deprivation. Specifically, both bacteria containing diets, either 7% B or 3.5% Y + 3.5% B, resulted in increased longevity for both sexes compared to the 7% Y fed flies. Interestingly, the males fed on 7% B diet lived longer than males fed on 3.5% Y + 3.5% B whereas the opposite effect was recorded for females. The interaction of diet treatment and sex was also significant (Wald’s t-test t = 7.90, df = 2, *P* = 0.019) indicating the different effect between full (7% B) and partial (3.5% Y + 3.5% B) yeast replacement on males and females (Fig. 10).

### Effect of brewer’s yeast replacement with *Enterobacter* sp. AA26 on males’ mating competitiveness

**Fig. 11:**
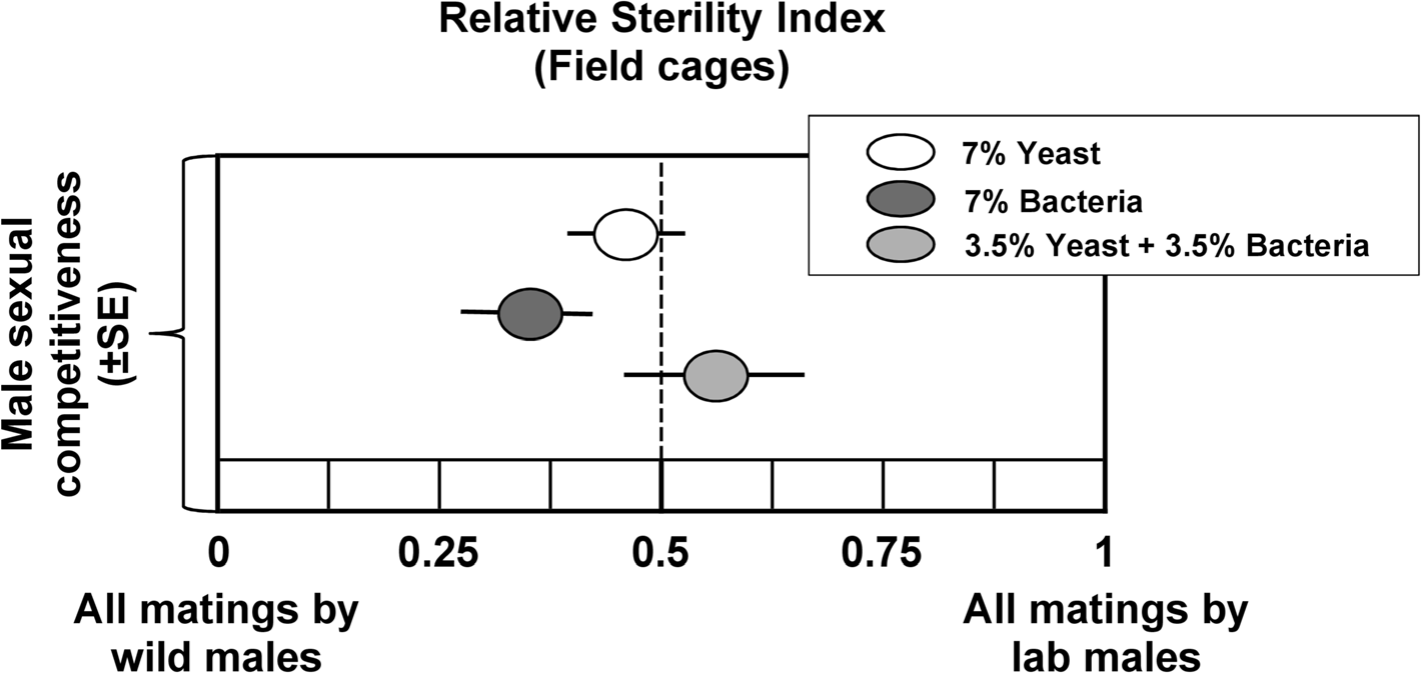
Mating competitiveness of males fed on the three protein source diets

Brewer’s yeast replacement with *Enterobacter* sp. AA26 biomass did not exert any significant effect on the mating competitiveness ability of irradiated Vienna 8 males when tested against wildish males and competing for ‘wildish’ females (F = 1.76, df = 2,18, *P* = 0.20). The Relative Sterility Index (RSI) for the three treatments tested (7% Y, 7% B, 3.5% Y + 3.5% B) are depicted in Fig. 11.

## Discussion

Our results demonstrate that *Enterobacter* sp. AA26 is an adequate nutritional source for *C. capitata* larvae, comprising an effective substitute for brewer’s yeast. Incorporating inactive bacterial cells in the larval diet conferred a number of substantial beneficial effects on medfly biology. The consumption of bacteria-based diet (either as full or partial yeast replacement) resulted in decreased immature stages mortality, accelerated immature development, increased pupal weight, and elongated the survival under stress conditions. Moreover, neither the partial nor the complete replacement of yeast with *Enterobacter* sp. AA26 had significant impact on adult sex ratio, females’ fecundity, adults’ flight ability and males’ mating competitiveness. The absence of both yeast and *Enterobacter* sp. AA26 (deprivation of protein source and possible other important nutrients) from the larval diet detrimentally affected the larval development, survival and elongated the immature developmental duration.

In medfly, the currently used rearing protocols largely rely on yeast, which consists of the main protein source in the larva artificial diets. The critical role of yeast provision on the larval development and the expression of desirable biological and behavioral traits have been pointed out by several previous studies [33, 35–46], whereas no reliable alternative protein source for medfly mass-rearing has been reported. The idea of exploiting the *Enterobacter* sp. AA26 biomass as the main protein source for medfly stemmed from the structural composition of the bacterial cells, which contain notable amount of protein/essential amino-acids (53.7 ± 1.2% protein, *n* = 3) [47].

The evaluation of the different diet formulations revealed the comparative advantage of the *Enterobacter* sp. AA26-based diets on several medfly fitness parameters. Both partial and full replacement of brewer’s yeast with *Enterobacter* sp. AA26 bacteria conferred substantial reduction on immature developmental duration and immature stages mortality. Moreover, the beneficial effects of the *Enterobacter* sp. AA26-based diets were not only restricted to developmental related parameters. Testing the adult performance, we showed that the consumption of bacterial cells during larval stage increased the adults’ longevity under stress conditions. This result confirms Yuval et al. (1998), who noted that the quality of the larval diet could be reflected in adult nutritional reserves and fitness traits [36]. The magnitude of the beneficial effects of brewer’s yeast replacement differed between the two bacterial treatments (full and the partial replacement). For example, whereas the accelerated immature development was more pronounced on the full replacement treatment, the reduction of immature mortality was more distinct on the partial replacement, indicating the different biological value of yeast and bacteria as protein sources. It is noteworthy that the bacteria exploitation as protein source did not impose any kind of inferiority to the produced adults. Given that our experiments were conducted with Vienna 8 D53^−^ GSS, a medfly strain that is constantly reared on yeast and therefore adapted to such a diet, the creation of a parallel Vienna 8 GSS line for rearing in bacterial diet could be a quite promising perspective. Allowing the adaptation to the new dietary environment and following up the assessment of the same biological traits after a few generations could possibly reveal even better results, rendered by the optimization of the symbiotic fauna or the natural selection of the most suitable genetic characters [3, 48–50].

The investigation of the role of intestinal bacteria on medfly’s biology has been a popular scientific field over the last years, particularly since Coronado-Gonzales et al. (2008) [51] confirmed that medfly and some other tephritids are adapted to bacteria as protein sources in view that their mouthparts only allow the ingestion of liquids and suspended particles less than 0.5 μm in size, such as bacteria of the *Enterobacteriaceae*. In contrast with our study where *Enterobacter* sp. AA26 was considered as a substantial component of the insect diet and a potential brewer’s yeast substitute, all the previous research used gut bacteria only as a supplementary additive of the existing, yeast-based, diets. Most of these efforts aimed to explore the probiotic role of *Enterobacteriaceae*, the most common microbial taxa present in medfly gut [6, 10, 52]. Indeed, “live” bacteria provision conferred substantial improvement of several biological and behavioural traits of *C. capitata*. The decreased mortality rates in immature stages, the accelerated developmental duration, the improved flight ability and the improved males’ sexual performance (mating latency time) were the most pronounced beneficial effects attributed to the “live” bacteria incorporation in medfly dietary. The hypothesis that the consumption of “live” bacteria enables their colonization and propagation in the gut lumen could possibly explain the latter outcomes. However, it is evidenced that even the “inactive” form of bacteria accounts for important medfly fitness traits when used as diet supplement. Working with the Vienna 8 GSS strain, Augustinos et al. (2015) [14] and Kyritsis et al. (2017) [53] highlighted the reduction of immature developmental duration after addition of autoclaved bacterial cells in brewer’s yeast-based larval diet (*Enterobacter* sp. AA26 and *Klebsiella oxytoca*, respectively). Considering i) the relative low concentrations of “inactive” bacteria cells used in the aforementioned studies, and ii) their supplementary role in the yeast-based diet, the beneficial effects detected cannot be fully explained only by their nutritional role. In fact, recent studies in food science introduce the “inactive” bacteria as potential health-promoting agents (paraprobiotics) due to their potential interactions with the hosts’ immune system [54–57].

From an applied point of view, the optimization of the insects’ rearing protocols is a major concern in mass-rearing industries. Currently, the large quantities of yeast needed for medfly rearing can be provided by only a few suppliers and the price is determined under monopoly or oligopoly market conditions. It is indicative that at least 12% of the whole medfly production cost is allocated to the yeast-related expenses (purchase, shipment, storage), (Ramirez personal communication). In light of our results, it appears that *Enterobacter* sp. AA26 inactive cells probably fulfill the same or similar nutritional “pathways” with brewer’s yeast (a similar study with torula yeast should also be performed), representing a reliable alternative protein source for medfly. The potential and feasibility of mass-producing *Enterobacter* sp. AA26, either commercially or in mass-rearing facilities (with the associated cost of bacteria culture, recruitment of specialized personnel, compliance to health-related regulations) should be evaluated by detailed cost-benefit analyses. Beyond medfly, future studies should further explore the potential of using inactive bacteria as main protein source, as well as should investigate the importance of yeast, *Asaia* bacteria and perhaps other overlooked components of the larval gut microbiota [58, 59] in an attempt to reduce the costs of the mass-rearing and at the same time maintain or even improve the biological quality of other SIT candidate species.

## Conclusions

*Enterobacter* sp. AA26 dry biomass can fully replace the brewer’s yeast as a protein source in medfly larval diet without any effect on the productivity and the biological quality of reared medfly of VIENNA 8 GSS as assessed by the FAO/IAEA/USDA standard quality control tests.

## Supplementary information

**Additional file 1.** Daily allocation of the immature stages duration for the four protein source treatments.

## Data Availability

The datasets used and analyzed during the current study are available from the corresponding author on reasonable request.

## Abbreviations

AW-IPM: Area Wide Integrated Pest Management
B: Bacterial larval diet containing *Enterobacter* sp. AA26 biomass
FAO: Food and Agriculture Organization
GSS: Genetic Sexing Strain
IAEA: International Atomic Energy Agency
IPCL: Insect Pest Control Laboratory
SIT: Sterile Insect Technique
USDA: United States Department of Agriculture
Y: larval diet containing brewer’s yeast
