# Supplementary figures and images for: Enterobacter sp. AA26 gut symbiont as a protein source for Mediterranean fruit fly mass-rearing and sterile insect technique applications
Source: BMC Microbiol. 2019 Dec 24;19(Suppl 1):288. doi: 10.1186/s12866-019-1651-z (PMC6929400; doi:10.1186/s12866-019-1651-z)

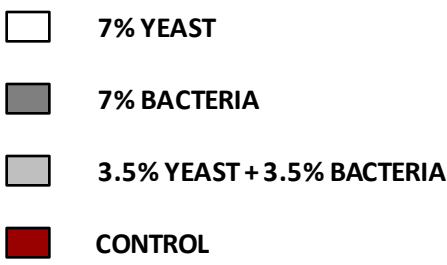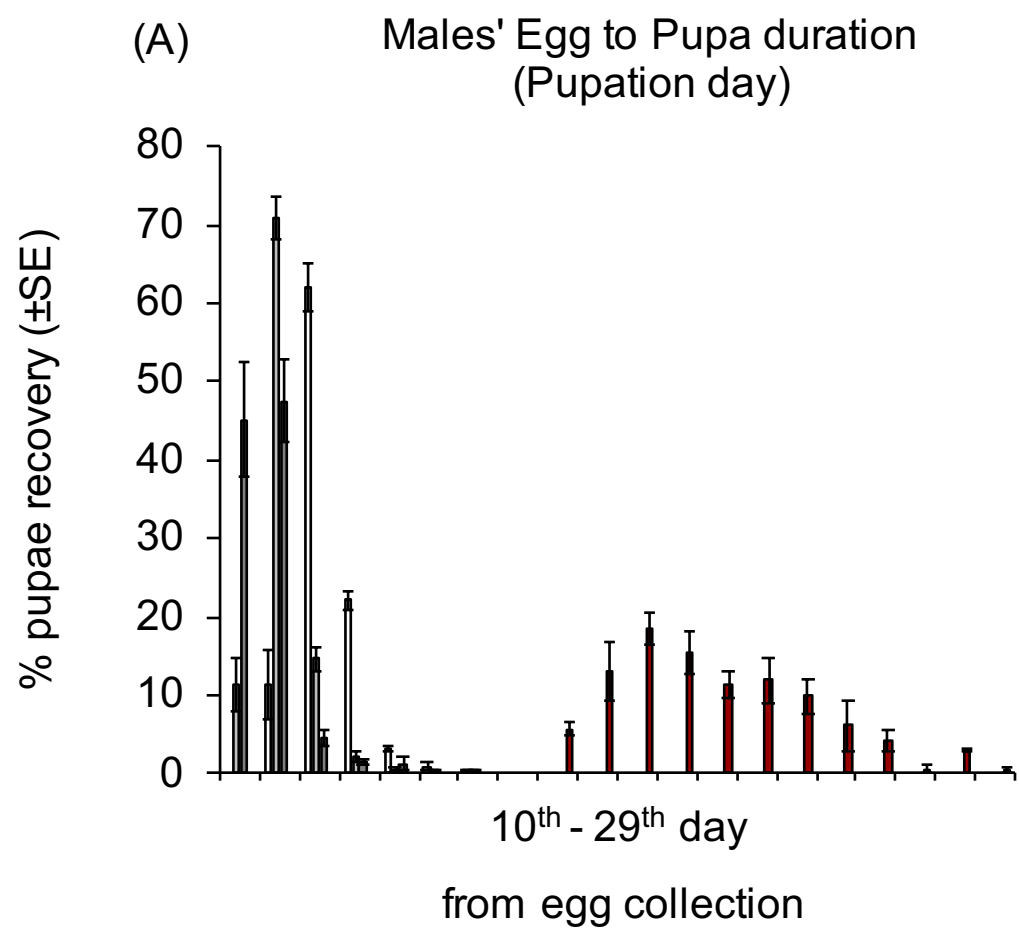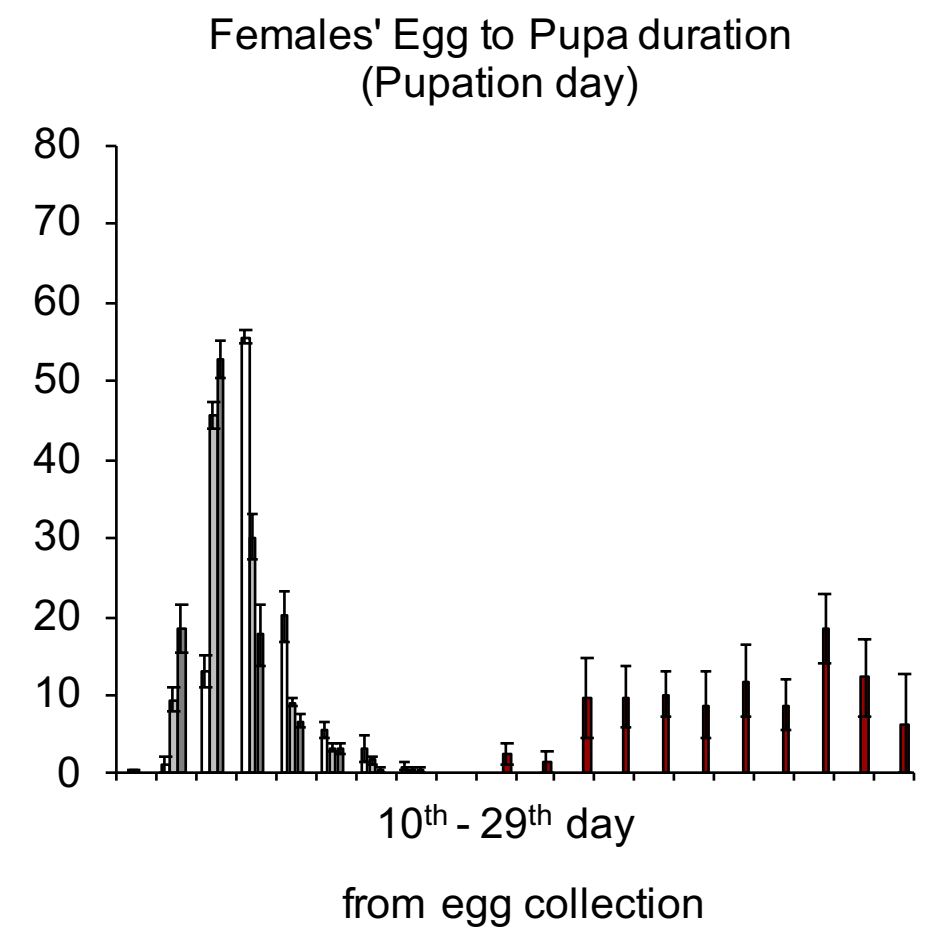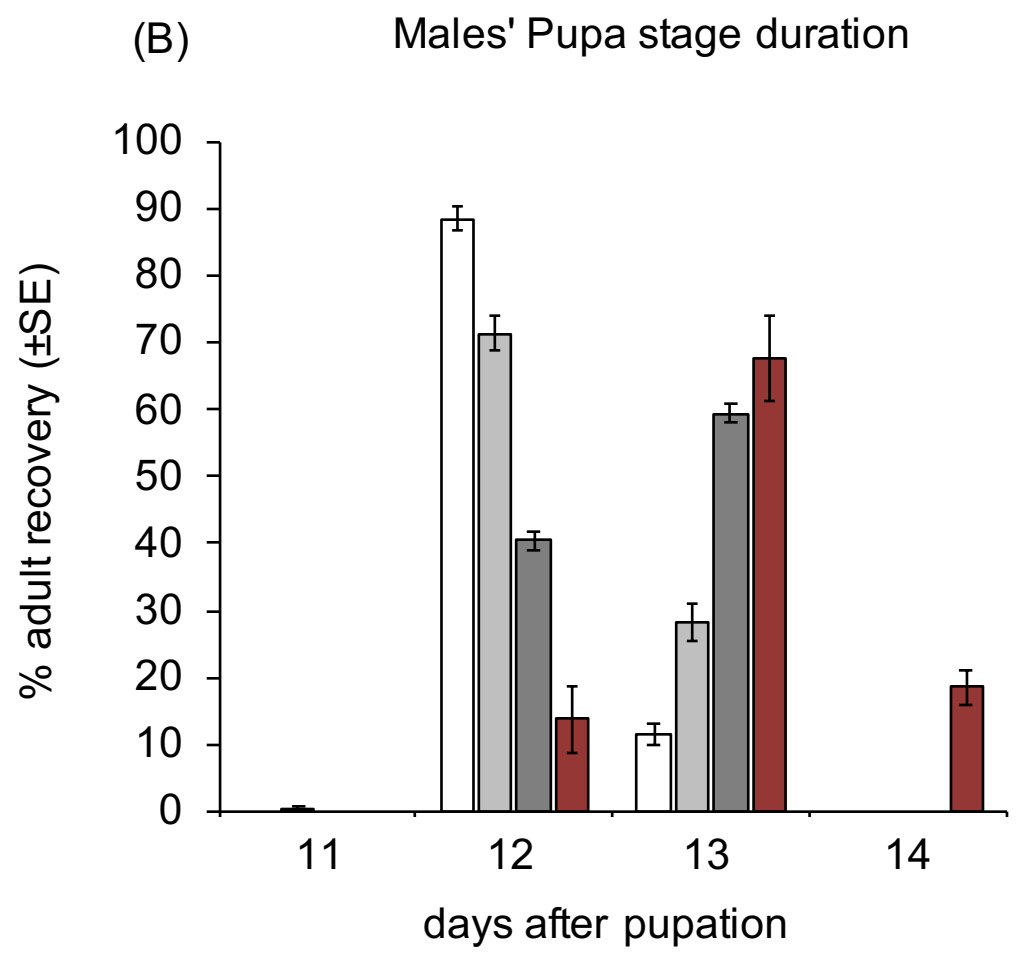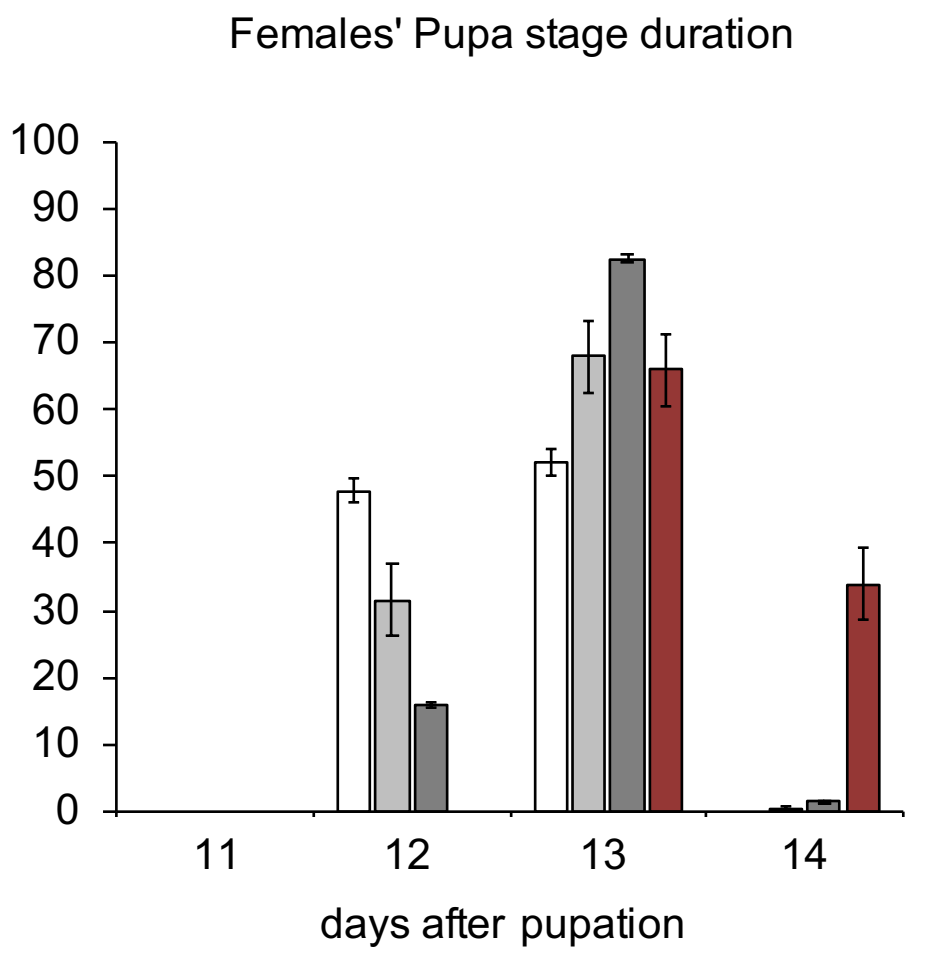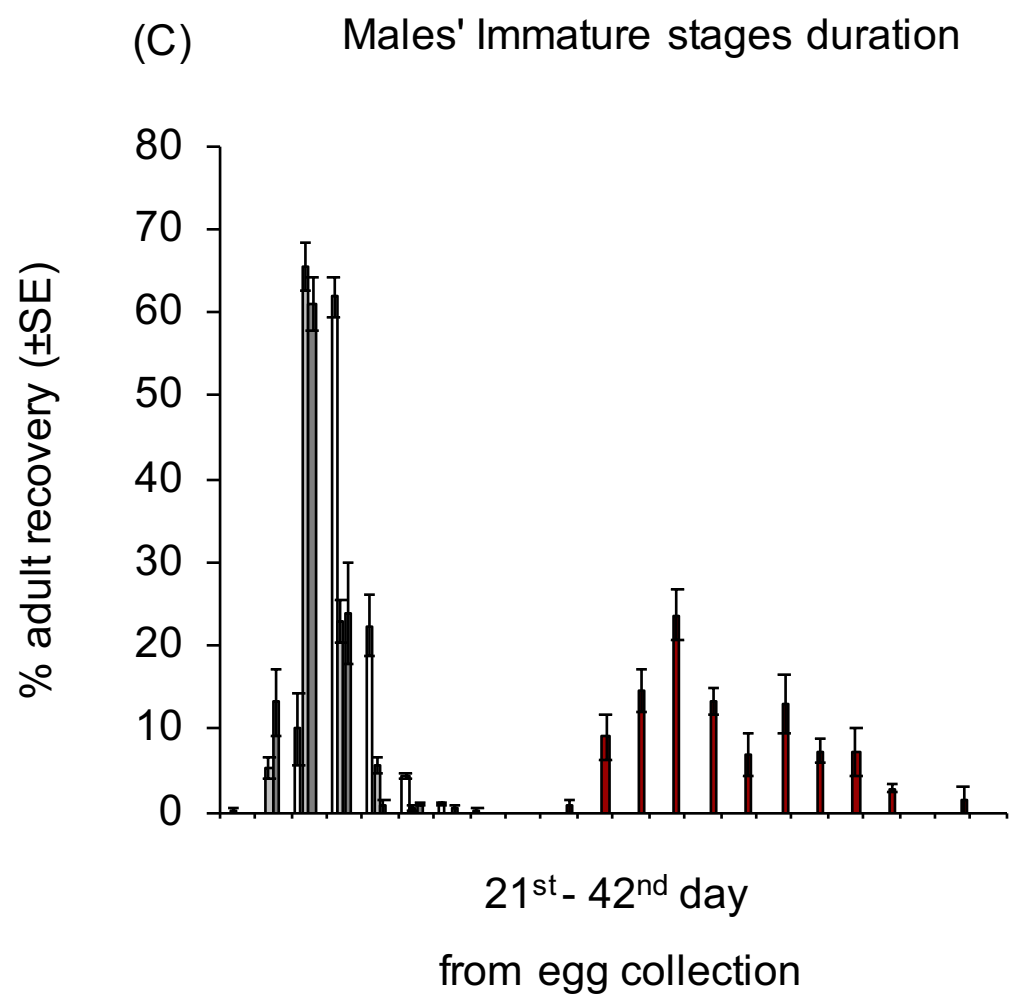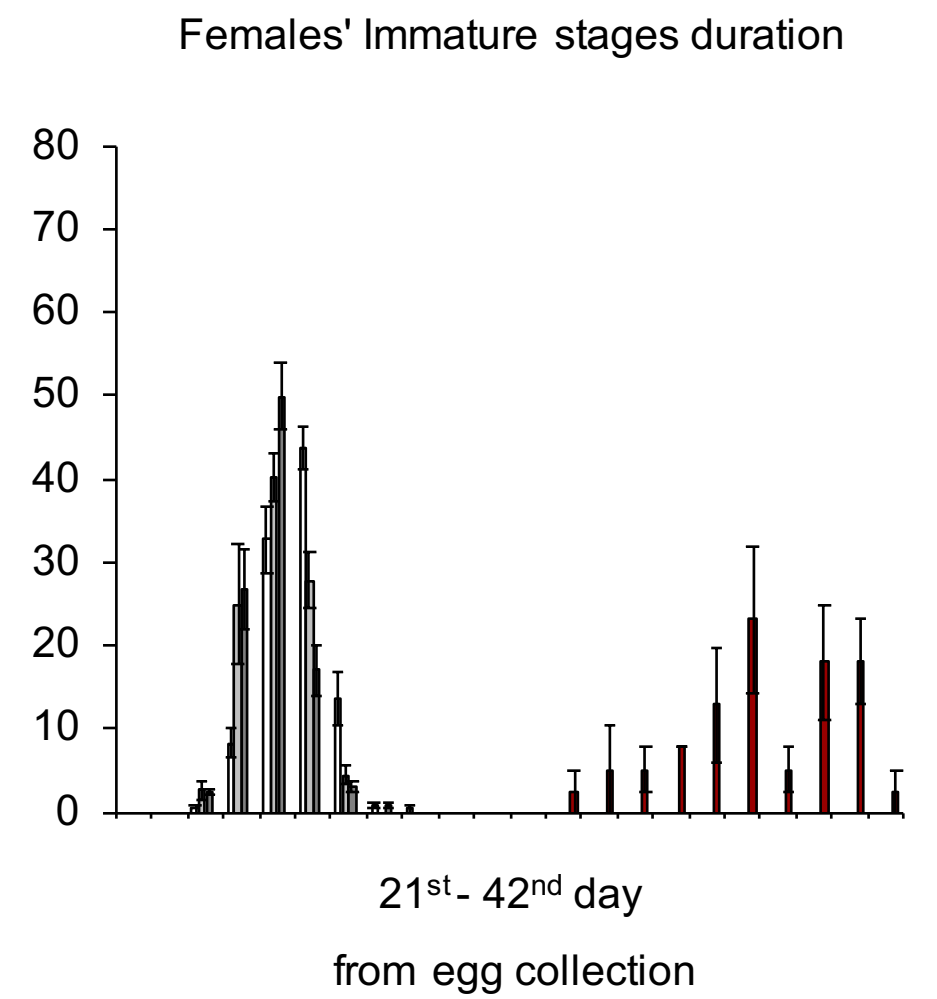

Supplement: Supplementary file 1 — Additional file 1. Daily allocation of the immature stages duration for the four protein source treatments. [file 12866_2019_1651_MOESM1_ESM.pdf]
